# Supplementary material for: Investigating CRISPR spacer targets and their impact on genomic diversification of Streptococcus mutans
Source: Front Genet. 2022 Sep 15;13:997341. doi: 10.3389/fgene.2022.997341 (PMC9522601; doi:10.3389/fgene.2022.997341)
Supplement: Supplementary file 1 [file DataSheet1.PDF]

Supplemental Material for:

Investigating CRISPR spacer targets and their impact on genomic diversification of *Streptococcus mutans*

Alejandro R. Walker[1] and Robert C. Shields[2*]
[1] Department of Oral Biology, University of Florida, Gainesville, Florida, USA
[2] Department of Biological Sciences, Arkansas State University, Jonesboro, Arkansas, USA

Supplementary Table S1. Bacteriophage targeted by *S. mutans* CRISPR spacers.

Phage strain	CRISPR spacer sequence	Target	Host
**Enterococcus phage vB_EfaS_IME197**	TATCTAACCTATCCTTACCTAACCTTACCT	replication initiation protein	*E. faecalis*
**Enterococcus phage vB_EfaS_IME197**	ATCTAACCTATCCTTACCTAACCTTACCTG	replication initiation protein	*E. faecalis*
**Lactobacillus phage LF1**	TCTAACCTATCCTTACCTAACCTTACCTG	replication initiation protein	*L. fermentum*
**Staphylococcus phage CNPH82**	TGTTTACCGTCATAATAGCAAGGTAAGAT	conserved phage protein	*S. epidermidis*
**Staphylococcus phage CNPx**	TGTTTACCGTCATAATAGCAAGGTAAGAT	conserved phage protein	*S. epidermidis*
**Staphylococcus phage PH15**	TGTTTACCGTCATAATAGCAAGGTAAGAT	conserved phage protein	*S. epidermidis*
**Staphylococcus phage vB_SepiS-philPLA5**	TGTTTACCGTCATAATAGCAAGGTAAGAT	conserved phage protein	*S. epidermidis*
**Staphylococcus phage vB_SepiS-philPLA7**	TGTTTACCGTCATAATAGCAAGGTAAGAT	conserved phage protein	*S. epidermidis*
**Streptococcus virus Sfi21**	AGAGAACTCGAAGAAAATACCGATAAGACA	orf123	*S. thermophilus*
**Streptococcus phage 7201**	TGGGTGCTAAAGGTGATGACTATCGTTTCA	orf33	*S. thermophilus*
**Streptococcus phage A25**	ACTTGCATATACACATTTTGTTCAACATCA	portal protein	*S. pyogenes*
**Streptococcus phage APCM01**	multiple spacers		*S. mutans*
**Streptococcus phage M102**	multiple spacers		*S. mutans*
**Streptococcus phage M102AD**	multiple spacers		*S. mutans*
**Streptococcus phage smHBZ8**	multiple spacers		*S. mutans*
**Streptococcus phage phiNJ2**	ACTTGCATATACACATTTTGTTCAACATCA	portal protein	*S. suis*
**Streptococcus phage T12**	TTATCAAGTTGTCCATACTGAGCTTTCATAATT	putative methyltransferase	*S. pyogenes*
**Streptococcus phage T12**	AATTATGAAAGCTCAGTATGGACAACTTGATAA	putative methyltransferase	*S. pyogenes*
**Streptococcus phage T12**	TATCAAGTTGTCCATACTGAGCTTTCATAATT	putative methyltransferase	*S. pyogenes*

Supplementary Table S2. *S. mutans* genes targeted by *S. mutans* CRISPR spacers.

Gene	Product	Core Genome	Evidence Self Target-targeting
ackA	Acetate kinase	Core	Yes
adeP	Adenine permease AdeP	Cloud	
aroA	3-phosphoshikimate 1-carboxyvinyltransferase	Core	Yes
atpD	ATP synthase subunit beta	Core	
cbl	HTH-type transcriptional regulator cbl	Core	Yes
clpC	ATP-dependent Clp protease ATP-binding subunit ClpC	Core	
clpL	ATP-dependent Clp protease ATP-binding subunit ClpL	Cloud	

clpP1	ATP-dependent Clp protease proteolytic subunit 1	Cloud	
clpX	ATP-dependent Clp protease ATP-binding subunit ClpX	Core	Yes
coaD	Phosphopantetheine adenylyltransferase	Core	Yes
comEC	ComE operon protein 3	Shell	Yes
dpnM	Modification methylase DpnIIA	Cloud	
ebh	Extracellular matrix-binding protein ebh	Cloud	Yes
esaA	ESAT-6 secretion accessory factor EsaA	Cloud	Yes
fccA	Fumarate reductase flavoprotein subunit	Shell	
fhaB	Filamentous hemagglutinin	Cloud	Yes
fieF	Ferrous-iron efflux pump FieF	Core	
fruA	Fructan beta-fructosidase	Core	
glnR	HTH-type transcriptional regulator GlnR	Core	Yes
grsB	Gramicidin S synthase 2	Shell	Yes
gtfC	Glucosyltransferase-SI	Shell	Yes
hcaR	Hca operon transcriptional activator HcaR	Cloud	
hin	DNA-invertase hin	Cloud	
hisS	Histidine--tRNA ligase	Core	Yes
Int-Tn	Transposase from transposon Tn916	Shell	
lacF	Lactose transport system permease protein LacF	Core	
lgrD	Linear gramicidin synthase subunit D	Shell	
lpxD	UDP-3-O-(3-hydroxymyristoyl)glucosamine N-acyltransferase	Cloud	
ltaS2	Lipoteichoic acid synthase 2	Core	Yes
ltrA	Group II intron-encoded protein LtrA	Cloud	
mhbT	3-hydroxybenzoate transporter MhbT	Shell	Yes
mleA	Malolactic enzyme	Shell	Yes
mrp	Iron-sulfur cluster carrier protein	Core	Yes
nfr1	NADH-dependent flavin reductase subunit 1	Soft Core	Yes
nrdG	Anaerobic ribonucleoside-triphosphate reductase-activating protein	Cloud	
parE	DNA topoisomerase 4 subunit B	Core	Yes
pbuG	Guanine/hypoxanthine permease PbuG	Core	Yes
plsX	Phosphate acyltransferase	Core	Yes
ppsB	Plipastatin synthase subunit B	Shell	Yes
ppsC	Plipastatin synthase subunit C	Shell	Yes
pyrG	CTP synthase	Core	Yes
radD	Putative DNA repair helicase RadD	Cloud	Yes
recF	DNA replication and repair protein RecF	Core	
repA	Regulatory protein RepA	Cloud	
rlmCD	23S rRNA (uracil-C(5))-methyltransferase RlmCD	Soft Core	
rnmV	Ribonuclease M5	Soft Core	Yes

secA	Protein translocase subunit SecA	Core	Yes
smc	Chromosome partition protein Smc	Soft Core	
soj	Sporulation initiation inhibitor protein Soj	Cloud	
spaP	Cell surface antigen I/II	Shell	Yes
ssaA	Staphylococcal secretory antigen SsaA	Cloud	
ssb	Single-stranded DNA-binding protein	Core	
ssrA	transfer-messenger RNA%2C SsrA		Yes
tilS	tRNA(Ile)-lysidine synthase	Core	
topB	DNA topoisomerase 3	Cloud	
traG	Conjugal transfer protein TraG	Shell	
ubiE	Ubiquinone/menaquinone biosynthesis C-methyltransferase UbiE	Shell	
urdA	Urocanate reductase	Cloud	
valS	Valine--tRNA ligase	Shell	Yes
xerC	Tyrosine recombinase XerC	Shell	
xerD	Tyrosine recombinase XerD	Shell	
xre	HTH-type transcriptional regulator Xre	Cloud	
yhdJ	DNA adenine methyltransferase YhdJ	Cloud	
**(blank)**	hypothetical protein	n/a	Variable
**(blank)**	N-acetylmuramoyl-L-alanine amidase domain-containing protein	Shell	Yes
**(blank)**	putative ABC transporter ATP-binding protein		
**(blank)**	putative cation efflux system protein		Yes
**(blank)**	Putative multidrug export ATP-binding/permease protein		Yes
**(blank)**	putative multidrug-efflux transporter		
**(blank)**	Ribonuclease		

Supplementary Table S3. *S. mutans* strains that carry self-targeting CRISPR spacers.

Strain	Self-target gene	Self-target product	Contains Acr Protein
**smu125**	nfr1_2	NADH-dependent flavin reductase subunit 1	
**smu173**		hypothetical protein	Yes
**smu174**	ssrA	transfer-messenger RNA%2C SsrA	
**smu179**	gtfC_6	Glucosyltransferase-SI	
**smu179**	gtfC_6	Glucosyltransferase-SI	
**smu179**	gtfC_6	Glucosyltransferase-SI	
**smu179**	lacF_2	Lactose transport system permease protein LacF	
**smu179**		hypothetical protein	
**smu179**		hypothetical protein	
**smu181**	ackA	Acetate kinase	
**smu184**	valS	Valine--tRNA ligase	
**smu185**	valS_1	Valine--tRNA ligase	
**smu185**	valS_2	Valine--tRNA ligase	

**smu187**	valS	Valine--tRNA ligase	
**smu192**	lgrD_2	Linear gramicidin synthase subunit D	
**smu193**	lgrD_2	Linear gramicidin synthase subunit D	
**smu194**	lgrD_2	Linear gramicidin synthase subunit D	
**smu201**		hypothetical protein	
**smu209**	nfr1_1	NADH-dependent flavin reductase subunit 1	
**smu216**	secA	Protein translocase subunit SecA	
**smu217**	gtfC_1	Glucosyltransferase-SI	
**smu224**	rnmV	Ribonuclease M5	
**smu225**	rnmV_1	Ribonuclease M5	
**smu238**	mleA	Malolactic enzyme	
**smu240**	secA	Protein translocase subunit SecA	
**smu251**	aroA	3-phosphoshikimate 1-carboxyvinyltransferase	
**smu254**		hypothetical protein	
**smu262**	cbl	HTH-type transcriptional regulator cbl	
**smu263**	rnmV	Ribonuclease M5	
**smu267**	rnmV	Ribonuclease M5	
**smu268**	rnmV_1	Ribonuclease M5	
**smu281**	mleA	Malolactic enzyme	
**smu283**	secA	Protein translocase subunit SecA	
**smu294**	aroA	3-phosphoshikimate 1-carboxyvinyltransferase	
**smu297**		hypothetical protein	
**smu305**	cbl	HTH-type transcriptional regulator cbl	
**smu306**	rnmV	Ribonuclease M5	
**smu315**	mleA	Malolactic enzyme	
**smu318**		hypothetical protein	
**smu320**		hypothetical protein	
**smu324**	grsB_1	Gramicidin S synthase 2	
**smu324**	rlmCD_2	23S rRNA (uracil-C(5))-methyltransferase RlmCD	
**smu324**	rlmCD_2	23S rRNA (uracil-C(5))-methyltransferase RlmCD	
**smu327**	hisS	Histidine--tRNA ligase	
**smu327**		hypothetical protein	
**smu327**		hypothetical protein	
**smu327**		hypothetical protein	
**smu327**		hypothetical protein	
**smu329**	hisS	Histidine--tRNA ligase	
**smu332**		hypothetical protein	
**smu333**		hypothetical protein	
**smu335**		putative ABC transporter ATP-binding protein	
**smu336**	radD	Putative DNA repair helicase RadD	
**smu337**	spaP_2	Cell surface antigen I/II	
**smu337**		hypothetical protein	
**smu338**	cbl	HTH-type transcriptional regulator cbl	
**smu34**	parE	DNA topoisomerase 4 subunit B	

**smu340**	cbl	HTH-type transcriptional regulator cbl	
**smu341**	cbl	HTH-type transcriptional regulator cbl	
**smu343**	fccA_2	Fumarate reductase flavoprotein subunit	
**smu344**		hypothetical protein	
**smu345**	fccA_2	Fumarate reductase flavoprotein subunit	
**smu349**		hypothetical protein	
**smu362**	fruA_2	Fructan beta-fructosidase	
**smu371**	pbuG	Guanine/hypoxanthine permease PbuG	
**smu372**	fhaB	Filamentous hemagglutinin	
**smu372**	fhaB	Filamentous hemagglutinin	
**smu375**	esaA_2	ESAT-6 secretion accessory factor EsaA	Yes
**smu375**	smc_6	Chromosome partition protein Smc	Yes
**smu376**	esaA_2	ESAT-6 secretion accessory factor EsaA	Yes
**smu376**	smc_6	Chromosome partition protein Smc	Yes
**smu378**	coaD	Phosphopantetheine adenylyltransferase	
**smu378**	ppsB	Plipastatin synthase subunit B	
**smu382**	atpD	ATP synthase subunit beta	
**smu383**		hypothetical protein	
**smu39**	pyrG	CTP synthase	
**smu390**	ppsC	Plipastatin synthase subunit C	
**smu391**	ppsC	Plipastatin synthase subunit C	
**smu395**		hypothetical protein	
**smu401**	ebh	Extracellular matrix-binding protein ebh	
**smu401**		hypothetical protein	
**smu409**	smc_2	Chromosome partition protein Smc	
**smu410**		hypothetical protein	
**smu411**		hypothetical protein	
**smu411**		hypothetical protein	
**smu412**	nfr1_2	NADH-dependent flavin reductase subunit 1	
**smu414**		hypothetical protein	Yes
**smu416**	comEC	ComE operon protein 3	
**smu416**		hypothetical protein	
**smu419**		hypothetical protein	Yes
**smu422**	mhbT	3-hydroxybenzoate transporter MhbT	
**smu422**	mhbT	3-hydroxybenzoate transporter MhbT	
**smu423**	clpX	ATP-dependent Clp protease ATP-binding subunit ClpX	
**smu423**	ppsC	Plipastatin synthase subunit C	
**smu426**		hypothetical protein	Yes
**smu431**	ltaS2	Lipoteichoic acid synthase 2	
**smu445**	glnR	HTH-type transcriptional regulator GlnR	
**smu445**	lgrD_2	Linear gramicidin synthase subunit D	
**smu445**		hypothetical protein	
**smu454**		hypothetical protein	
**smu472**	mrp	Iron-sulfur cluster carrier protein	

**smu6**		hypothetical protein	
**smu6**		putative cation efflux system protein	
**smu76**	plsX	Phosphate acyltransferase	
